# BMP-7 induces apoptosis in human germinal center B cells and is influenced by TGF-β receptor type I ALK5

**DOI:** 10.1371/journal.pone.0177188

**Published:** 2017-05-10

**Authors:** Lise K. Bollum, Kanutte Huse, Morten P. Oksvold, Baoyan Bai, Vera I. Hilden, Lise Forfang, Sun Ok Yoon, Sébastien Wälchli, Erlend B. Smeland, June H. Myklebust

**Affiliations:** 1 Department of Cancer Immunology, Institute for Cancer Research, the Norwegian Radium Hospital, Oslo, Norway; 2 Center for Cancer Biomedicine, University of Oslo, Oslo, Norway; 3 Faculty of Medicine, University of Oslo, Oslo, Norway; 4 Laboratory of Cellular Immunology, Ochsner Clinic Foundation, New Orleans, Louisiana, United States of America; 5 Transplantation Research Institute, Seoul National University College of Medicine, Seoul, South Korea; 6 Department of Cellular Therapy, the Norwegian Radium Hospital, Oslo, Norway; Augusta University, UNITED STATES

## Abstract

Selection and maturation of B cells into plasma cells producing high-affinity antibodies occur in germinal centers (GC). GCs form transiently in secondary lymphoid organs upon antigen challenge, and the GC reaction is a highly regulated process. TGF-β is a potent negative regulator, but the influence of other family members including bone morphogenetic proteins (BMPs) is less known. Studies of human peripheral blood B lymphocytes showed that BMP-6 suppressed plasmablast differentiation, whereas BMP-7 induced apoptosis. Here, we show that human naïve and GC B cells had a strikingly different receptor expression pattern. GC B cells expressed high levels of BMP type I receptor but low levels of type II receptors, whereas naïve B cells had the opposite pattern. Furthermore, GC B cells had elevated levels of downstream signaling components *SMAD1* and *SMAD5*, but reduced levels of the inhibitory *SMAD7*. Functional assays of GC B cells revealed that BMP-7 suppressed the viability-promoting effect of CD40L and IL-21, but had no effect on CD40L- and IL-21-induced differentiation into plasmablasts. BMP-7-induced apoptosis was counteracted by a selective TGF-β type I receptor (ALK4/5/7) inhibitor, but not by a selective BMP receptor type I inhibitor. Furthermore, overexpression of truncated ALK5 in a B-cell line counteracted BMP-7-induced apoptosis, whereas overexpression of truncated ALK4 had no effect. BMP-7 mRNA and protein was readily detected in tonsillar B cells, indicating a physiological relevance of the study. Altogether, we identified BMP-7 as a negative regulator of GC B-cell survival. The effect was counteracted by truncated ALK5, suggesting greater complexity in regulating BMP-7 signaling than previously believed.

## Introduction

Germinal centers (GC) are specialized structures that form transiently in secondary lymphoid organs following exposure to T cell-dependent antigens, and are the sites of B-cell maturation and selection. In the GC, the B cells’ immunoglobulin (Ig) genes undergo somatic hypermutation (SHM) and class switch recombination (CSR) to improve the affinity for antigens and gain specific effector function [1, 2]. SHM and CSR are complex processes initiated by activation-induced cytidine deaminase (AID) [3]. GC B cells are pre-programmed to undergo apoptosis due to the lack of anti-apoptotic factors, including Bcl-2 [2, 4, 5], and increased expression of apoptosis-inducing genes are dependent on a carefully regulated environment of cytokines [4]. IL-21, produced by T follicular helper (T_FH_) cells, exerts a powerful role in promoting differentiation to plasma cells and to induce CSR [6–9]. Together with follicular dendritic cells (FDC), T_FH_ cells provide support for GC B cells, also in the form of ligands for important co-receptors such as CD40 [10, 11]. In addition, inhibitory cytokines also participate in regulating the GC reaction. The suppressive role of transforming growth factor-β (TGF-β) in B cells is well known, and TGF-β signaling has been shown to be a strong inhibitor of cell growth and to inhibit production of IgM and IgG, but to promote IgA CSR [12–14].

The TGF-β family is a large superfamily of cytokines involved in regulation of proliferation, differentiation and apoptosis in different tissues and developmental stages. One subgroup of the TGF-β superfamily is the bone morphogenetic protein (BMP) subfamily containing 21 different cytokines [15]. BMP signaling is initiated when a BMP dimer binds to the BMP receptor complex, a heterotetramer comprised of type I (Activin receptor-like kinase 2 (ALK2), ALK3 and ALK6) and type II (BMPRII, Activin A receptor type IIA (ActRIIa) and ActRIIb) transmembrane serine/threonine receptor kinases [16, 17]. Furthermore, some recent studies suggest that BMPs may also utilize TGF-β receptors [18–20]. Receptor activation is followed by phosphorylation of the receptor-regulated Smad 1/5/8 which form a trimer with Smad 4 and translocate to the nucleus [16, 21]. The complex is joined by several cofactors which lead to transcription of target genes, including *ID1*, *ID2* and *ID3* [22].

Various BMPs can affect B-cell development at different stages [23]. BMP-4 has been shown to be a critical regulator of hematopoiesis [24–28], whereas BMP-6 inhibited proliferation of early B-progenitor cells as well as mature peripheral blood B cells [29, 30] and plasma cells [31–33]. Furthermore, BMP-2, -4, -6 and -7 reduced CD40L- and IL-21-induced Ig production in human naïve and memory B cells from peripheral blood [34]. The mechanism for BMP-induced suppression differed between the BMPs, as BMP-6 potently inhibited plasma-cell differentiation, whereas BMP-7 mainly induced apoptosis under the same conditions [34]. However, a detailed characterization of BMP effects in human B cells undergoing GC reaction has not been done. Here, we characterized the expression of BMP-signaling components and BMP-induced functional effects in various B-cell subsets from human tonsils, including GC B cells. Our work identified BMP-7 as a negative regulator of GC B-cell survival, hence adding further complexity to the process of regulatory mechanisms in B cells undergoing GC reaction.

## Materials and methods

### Human samples and cell lines

Tonsils were obtained from Agroklinikken (Asker, Norway), with written informed consent in accordance with the Declaration of Helsinki and the Regional Committees for Medical and Health Research Ethics, Region Eastern Norway (approved protocol REK#2010/1147a). The tonsils were processed to single cell suspension by mincing and stored as aliquots in liquid nitrogen. Peripheral blood was collected from anonymous, healthy donors at The Blood Bank in Oslo, after informed consent and with approval from regional authorities (REK S-03280). The human cell lines Jurkat, Sudhl-6 and Mino were from DSMZ (ACC 282, ACC 572 and ACC 687). The HK cell line was a kind gift from Dr. Choi, Ochsner Clinic Foundation, New Orleans, USA. All cell lines were sustained in RPMI 1640 (PAA Laboratories) supplemented with 10% fetal calf serum (FCS) and streptomycin / penicillin (PAA)), but Mino cells were cultured in X-VIVO 15 serum free media (Lonza, Switzerland) during functional studies.

### Reagents

The following primary antibodies were used: biotinylated anti-ActRIIa (BAF340), -ActRIIb (BAF339), -BMPRII (BAF811),—ALK2 (BAF637), -ALK3 (BAF820), -ALK4 (BAF222) -ALK5 (BAF 3025), -ALK6 (BAF505), biotinylated goat IgG (BAF108) (R&D Systems, MN, USA). The following antibodies were from BD Biosciences (NJ, USA): Anti-active caspase-3 Alexa647 (Clone: C92-605), anti-CD20 APC-H7 (Clone: L27), -IgD PerCP-Cy5.5 (Clone: IA6-2), -CD38 PerCP-Cy5.5 (Clone: HIT2), -CD38 PE-Cy7 (Clone: HB7), -CD38 FITC (Clone: HIT2), -CD3 V500 (Clone: UCHT1), -CD27 APC (Clone: M-T2701), -CD44 APC (Clone: G44-26), and biotinylated anti-CD44 (Clone: G44-26). Anti-IgD FITC (polyclonal, #F0189), -CD3 PE (Clone: UCHT1) and CD3 FITC (Clone: UCHT1) (Dako Glostrup, Denmark), anti-p-Smad 1/5 PE (Clone: D5B10), -p-Smad 2/3 PE (Clone: D27F4) and -p-ERK Alexa 647 (Clone: 197G2), -Smad 1 (#9743S), -Smad 4 (#9515) (Cell Signaling Technology, MA, USA), biotinylated anti-CD38 (Clone: HIT2) (eBioscience, CA, USA), anti-β-actin (clone: I-19) (Santa Cruz Biotechnology), and anti-BMP-7 (LSBio, #LS-C293046). The following secondary antibodies were used: rabbit anti-GAPDH (#100118; GeneTex, Irvine, CA). Biotinylated antibodies were detected using Streptavidin PE or Streptavidin APC (BD Biosciences, NJ, USA). CD40L and Enhancer for Ligands (ALX-850-064) were acquired from Alexis Biochemicals, Enzo Life Sciences (NY, USA). IL-21 (PHC0214) was purchased from Invitrogen (CA, USA) and from eBioscience. BMP-2, BMP-4, BMP-6 and Activin A were purchased from R&D Systems (MN, USA). BMP-7 was purchased from R&D Systems and from BioLegend (CA, USA). The ALK2/ALK3 inhibitor LDN193189 (#S2618) and the ALK4/ALK5/ALK7 inhibitor SB431542 (#S1067) were purchased from Selleckchem (TX, USA).

The following probes were from Applied Biosciences (CA, USA): *SMAD1* FAM (Hs00195432_m1), *SMAD5* FAM (Hs00195437_m1), *SMAD4* FAM (Hs00929647_m1), *SMAD6* FAM (Hs00178579_m1), *SMAD7* FAM (Hs00998193_m1) and *PGK-1* VIC (4326318E), *1D1* (Hs03676575_s1), *ID2* (Hs04187239_m1), *ID3* (Hs00954037_g1), GAPDH FAM (4352934E), BMP7 FAM (Hs00233476_m1, Hs00233477_m1 and Hs01002399_m1), PGK-1 FAM (4333765F).

### Cell culture conditions

Tonsillar GC B cells, naïve and memory B cells (each at 1 x 10^5^ cells/ml) were cultured in 48-well plates containing irradiated HK cells seeded 24 hours in advance (1.2 x 10^3^ cells/well, 3000 rad). Primary B cells were cultured in RPMI with 10% FCS, together with rhCD40 ligand (CD40L) (Alexis Biochemicals) at 0.25 μg/ml (pre-incubated with an Enhancer for ligands (1 μg/ml; anti-FLAG antibody that crosslinks FLAG-tagged rhCD40L), according to manufacturer’s recommendations.

In addition, IL-21 was added as specified at 20 ng/ml in the presence or absence of BMP-2 (400 ng/ml), BMP-4 (25 ng/ml), BMP-6 (250 ng/mL) or BMP-7 (500 ng/mL) or as specified. RPMI with 10% FCS was used as culture medium, unless otherwise specified.

### Immunomagnetic bead isolation of B-cell subsets

Tonsillar B cells were isolated using negative isolation with CD3 Dynabeads (Thermo Fisher, MA, USA). CD3-depleted cells were split in two populations and incubated with antibodies for 10 min. CD44^-^ GC B cells were obtained through negative selection by combining Biotin Binder Dynabeads (Thermo Fisher, 50 μl beads per 10x10^6^ cells) with biotinylated anti-CD44 (BD Pharmingen) (1:50). A naïve/memory joint population was isolated using Biotin Binder Dynabeads (Thermo Fisher) with biotinylated anti-CD38 (eBioscience) (2.35 μg/mL). Cells and Dynabeads were incubated in the dark at 4°C with rotation. IgD-depleted memory B cells were obtained by negative selection by incubating CD19^+^ B cells with Pan Mouse IgG Dynabeads (Thermo Fisher) coated with mouse anti-human IgD Abs (BD) for 30 min at 4°C, followed by removal of beads.

### FACS analysis and cell sorting

Cells were labeled with antibodies and incubated at 4°C for 30 min. FACS analysis and sorting was carried out on a FACSCantoII and FACS Aria Flow Cytometer (BD), respectively. Using the anti-IgD FITC, -CD20 APC-H7, -CD27 APC, -CD3 PE and -CD38 PerCP-Cy5.5. B-cell subsets were obtained as specified: GC (CD20^+^IgD^-^CD27^-^CD3^-^CD38^+^), naïve (CD20^+^IgD^+^CD27^-^CD3^-^CD38^-^) and class switched memory B cells: (CD20^+^IgD^-^CD27^+^CD3^-^CD38^-^). Intracellular phospho-flow cytometry was performed on fixed cells (5 minutes incubation with 1.6% Paraformaldehyde (PFA), Electron Microscopy Sciences, PA, USA), permeabilized with 90% methanol and stained at room temperature for 60 min. All flow cytometry data were analyzed using the online Cytobank software (www.cytobank.org). Differences in protein expression are shown as arcsinh transformation, calculated as arcsinh (median fluorescence intensity (MFI) of activated cells/cofactor) subtracted by arcsinh (MFI of unstimulated cells/cofactor), using cofactors set to ≥ 150.

### Full length and truncated *TGFBR1* and *ACVRIB* constructs

To overexpress *TGFBR1 (ALK5)* and *ACVRIB (ALK4)*, we designed our constructs as previously done with *SMAD7* [35] where the gene of interest was cloned as a 2A-fusion with GFP, which generated one mRNA and two distinct proteins. All constructs were designed without STOP codon and, for *TGFBRI*, with an in frame *Kpn*I site in order to fuse them with 2A_GFP in a pENTR vector (Invitrogen) as previously described [35]. For *ACVRIB*, we created a 2A_mCherry construct in pENTR by amplifying mCherry and PCR-fusing it to 2A with a *Kpn*I site in the same frame as the GFP construct. This was done using the following primers: KI2AmCherr_F CAC CGG TAC CAG AGC CAA GAG AGG CAG CGG CGC CAC CAA CTT CAG CCT GCT GAA GCA GGC CGG CGA CGT GGA AGA GAA CCC TGG ACC AAT GGT GAG CAA GGG CGA G and mCherrSTOPERI_r TTG AAT TCT TAC TTG TAC AGC TCG TCC ATG. The full length sequence of human *TGFBR1 (ALK5)* and the truncated version (from the signal sequence to and excluding the GS domain to avoid positive signals, amino acid 1 to 174) were designed based on the NCBI Database sequence (accession number: NM_004612). These constructs were codon optimized and synthesized by Eurofins (Eurofins MWG Operon Ebersberg, Germany). The *ACVRIB* coding sequence was PCR extracted from pDONR223-ACVR1B which was a gift from William Hahn & David Root (Addgene plasmid # 23567). We subcloned the amplicon (truncated *ACVRIB*) using the InFusion kit (Clontech Laboratories Inc, CA, USA) with the following primers: (Alk4f) CCG CCC CCT TCA CCG ATG GCG GAG TCG GCC GGA G and tAlk4r (truncated) CCT CTC TTG GCT CTG gcC TTG TCT TTG GAG AGA C where the nucleotides in lower case are added to keep the frame of the fused vector (pENTR-mCherry). After sequence verification (Eurofins), the pENTR constructs were recombined to a Gateway compatible retroviral expression vector, pMP71-Gateway [36].

Viral particles were produced as described previously [36]. Briefly, Hek-P cells were transfected using Fugene-6 (Roche, Germany) with retroviral packaging vectors and the expression vector. After 24 hours of incubation at 37°C, medium was replaced with DMEM containing 1% FCS and cells were incubated at 32°C. Supernatants were harvested at 48 and 72 hours post transfection. Spinoculation of Mino cells was performed with 1 V of retroviral supernatant in a 12-well culture non-treated plate (Nunc, Roskilde, Denmark) pre-coated with retronectin (20 mg/mL, Takara Bio., Shiga, Japan). Two days later, cells were harvested with PBS-EDTA (0.5 mM), but cultured for at least 2 weeks before they were used in experiments.

### Viability and apoptosis assays

Viability was assessed by incubating cells with propidium iodide (PI, 5 μg/mL; Thermo Fisher). Apoptosis was measured on fixed cells (1.6% PFA) and permeabilized with methanol (≥ 90%), followed by staining with anti-active caspase-3 antibodies (Cell Signaling Technologies; 1:20 dilution). Terminal deoxynucleotidyl transferase dUTP nick end labeling (TUNEL) was determined by the In Situ Cell Death Detection Kit (Sigma-Aldrich, MO, USA) as described by the manufacturer. Samples were analyzed on FACS CantoII (BD).

### qPCR

RNA was isolated using RNeasy Plus kit (Qiagen, Hilden, Germany) and cDNA was synthesized using the Quantitect kit (Qiagen), adding 10 ng of total RNA per sample. Quantitative real-time PCR (qPCR) was carried out on a 7500 Real Time PCR System (Applied Biosciences). *SMAD* expression was measured in duplex (except *SMAD1*) with *PGK-1* VIC as endogenous control. Assays (see S1 Table for all probes) run in singleplex included *PGK-1* FAM as endogenous control. The relative expression levels were calculated using the ΔΔCT method, and normalized to human fetal brain total RNA from a 22 week old donor (BioChain, CA, USA), or from Sudhl-6 as specified.

### Western blot analysis

Cells were lysed and processed for SDS-PAGE. Mini Protean or Criterion TGX precast gels were used for SDS-PAGE (Bio-Rad Laboratories, CA, USA), transferred to PVDF membranes and hybridized with the indicated antibodies, followed by HRP-conjugated secondary antibodies. Protein bands were visualized using Amersham ECL Plus (GE Healthcare, Little Chalfont, UK) or Pierce ECL-2 (Thermo Fisher) with Hyperfilm (GE) or SuperSignal West Pico and Dura (Thermo Fisher Scientific) or Clarity (Bio-Rad) with Chemidoc MP (Bio-Rad) applied for imaging. Image processing was performed by use of ImageLab (Bio-Rad) and Quantity One software (BioRad).

### Immunocytochemistry

Naïve and GC B cells from tonsils were fixed in 4% PFA (Electron Microscopy Sciences, PA, USA) in PBS, washed in PBS and permeabilized in 90% methanol at -20°C. After washing in PBS, TUNEL staining (Sigma-Aldrich) was performed as described by the manufacturer, before cytospin samples were prepared using Shandon Cytospin 2 mounted in fluorescent mounting medium (Dako) containing Hoechst 33258 and visualized in a Zeiss LSM710 confocal unit (Carl Zeiss, Germany), equipped with a 25x/0.8 oil objective. All images were taken randomly from the Hoechst staining. Images were exported as tiff images and assembled in Illustrator (Adobe, CA, USA).

### Statistical analysis

Statistical significance was determined by applying either a two-tailed paired Student’s *t*-test or a two-tailed unequal variance Student’s *t*-test as specified. Statistical differences were corrected for multiple testing.

## Results

### Contrasting expression pattern of BMP receptors in GC and naïve B cells

**Fig 1:**
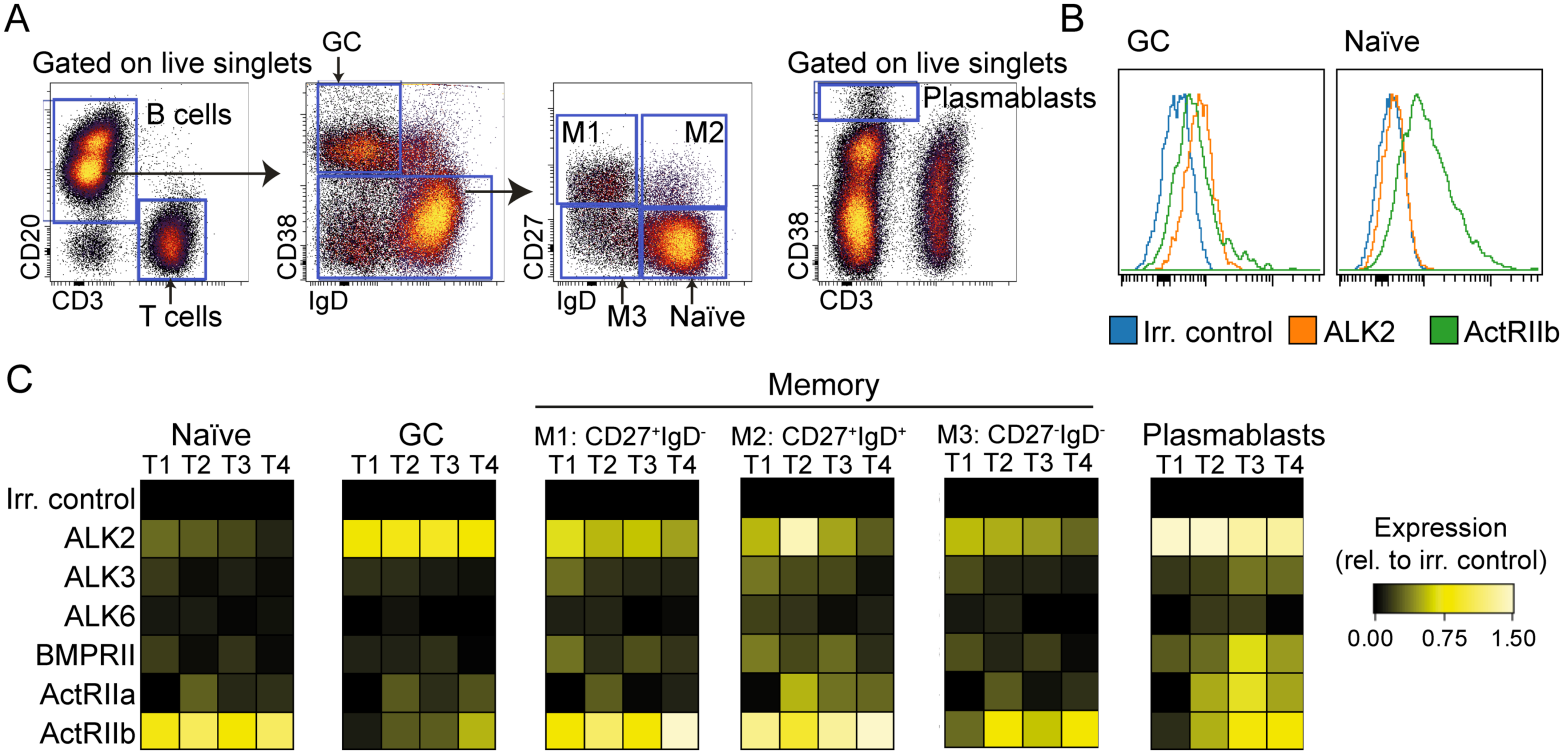
Tonsillar B-cell subsets have differential expression patterns of BMP receptors. Single-cell suspensions from human tonsils were stained with lineage markers and anti-BMP receptor antibodies, and analyzed by flow cytometry. (A) Gating strategy to identify B-cell subsets in tonsils: Naïve B cells were defined as CD3^-^CD20^+^CD38^-^IgD^+^CD27^-^, GC B cells as CD3^-^CD20^+^CD38^+^IgD^-^ and plasmablasts as CD3^-^CD38^hi^. Memory B cells were separated into three subsets: class switched CD20^+^CD3^-^CD38^-^IgD^-^CD27^+^, non-switched CD20^+^CD3^-^ CD38^-^IgD^+^CD27^+^ and class switched CD20^+^CD3^-^ CD38^-^IgD^-^CD27^-^ memory B cells. (B) Histogram overlays of receptor expression in GC and naïve B cells in donor T4. (C) Heatmaps of relative protein expression of BMP type I and type II receptors in tonsils from 4 different donors, denoted T1 –T4. The expression levels were normalized to irrelevant Ab control in each donor, using arcsinh transformation.

We have previously demonstrated expression of *BMP6* and *BMP7* mRNA in GC B cells [35]. In order to determine whether GC B cells are susceptible to the effects of BMPs, the expression of the different BMP receptors was determined by flow cytometry. Single cell suspensions from tonsils were stained with antibodies against BMP receptors type I (ALK2/ALK3/ALK6) or type II (BMPRII/ActRIIa/ActRIIb), combined with an antibody panel to identify GC B cells (CD3^-^CD20^+^CD38^+^IgD^-^), naïve B cells (CD3^-^CD20^+^CD38^-^IgD^+^CD27^-^), plasmablasts (CD3^-^CD38^hi^) and three distinct memory B-cell subsets (CD27^+^IgD^-^ class switched memory, CD27^+^IgD^+^ non-switched memory and CD27^-^IgD^-^ double negative memory B cells) (Fig 1A). This approach revealed a striking difference in receptor expression pattern between GC, naïve and memory subsets. GC B cells had high expression of ALK2, but low expression of type II receptors (Fig 1B and 1C). In contrast, naïve B cells showed low expression of ALK2, but high expression of ActRIIb, whereas the various memory B-cell subsets had high expression of both receptor subtypes (Fig 1B and 1C). Plasmablasts also showed high expression of ALK2 and usually expressed all type II receptors (Fig 1B and 1C). These results demonstrate that BMP receptor expression is differently regulated throughout B-cell maturation, suggesting variable sensitivity to BMPs.

### GC B cells express increased levels of Smad 1/5 and reduced levels of inhibitory *SMAD7*

**Fig 2:**
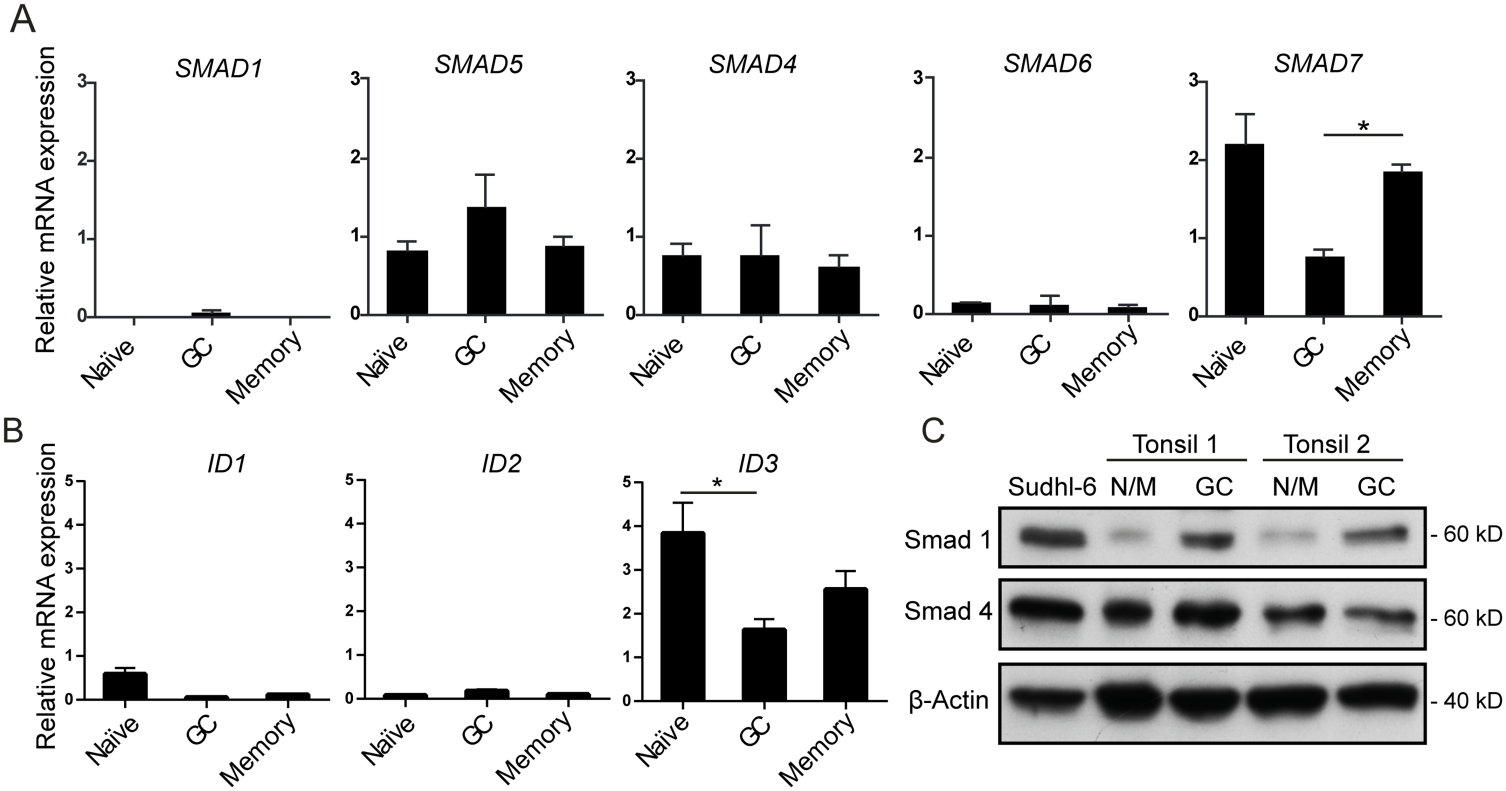
GC B cells have higher levels of r-Smads and lower levels of i-Smads as compared to naïve or memory B cells. mRNA expression was determined by qPCR of FACS-sorted B-cell subsets from tonsillar specimens. (A) *SMAD* mRNA expression and (B) *ID* mRNA expression. Relative mRNA expression is shown relative to PGK-1 as endogenous control and normalized to total RNA from human fetal brain. Shown is mean values ± SEM, *n* = 3. (C) Protein levels of Smads were determined by western blotting of B-cell subsets obtained by immunomagnetic bead separation of tonsil single-cell suspensions. Sudhl-6 cells were included as positive control and β-actin was used as loading control. N/M denotes naïve/memory B cells. *p < 0.05, paired two-tailed Student’s *t*-test.

To investigate whether GC B cells expressed Smad signaling components, we determined relative mRNA levels in FACS-sorted B-cell subsets by qPCR. GC, naïve and memory B cells were separated, using the gating strategy in Fig 1A. GC B cells expressed a 1.5-fold higher level of *SMAD5* than naïve B cells, and also expressed *SMAD1* which was undetectable in naïve and memory B cells (Fig 2A). *SMAD4* and *SMAD6* were expressed at similar levels in all subsets. In contrast, the inhibitory *SMAD7* was significantly lower in GC B cells than memory B cells (Fig 2A, *p* < 0.025). Naive, memory and GC B cells had detectable levels of *ID1*, *ID2* and *ID3* genes, and *ID3* was significantly higher in naive B cells than GC B cells (Fig 2B).

To validate these results at the protein level, we developed a strategy using two rounds of negative depletion with immunomagnetic beads to obtain “untouched” GC B cells and a combined population of naïve and memory B cells, which gave highly pure populations (> 88%) and higher cell number yields than FACS sorting (S1 Fig). Western blot analysis confirmed higher Smad 1 protein expression in GC B cells as compared to naïve/memory cells and no difference in Smad 4 protein levels (Fig 2C). Taken together, GC B cells had a different BMP receptor expression pattern than other B-cell subtypes and expressed higher levels of Smad1/5, but lower level of inhibitory *SMAD7*, suggesting that GC B cells might respond differently to BMPs.

### BMP-7 induces apoptosis, but does not affect plasmablast differentiation of GC B cells

**Fig 3:**
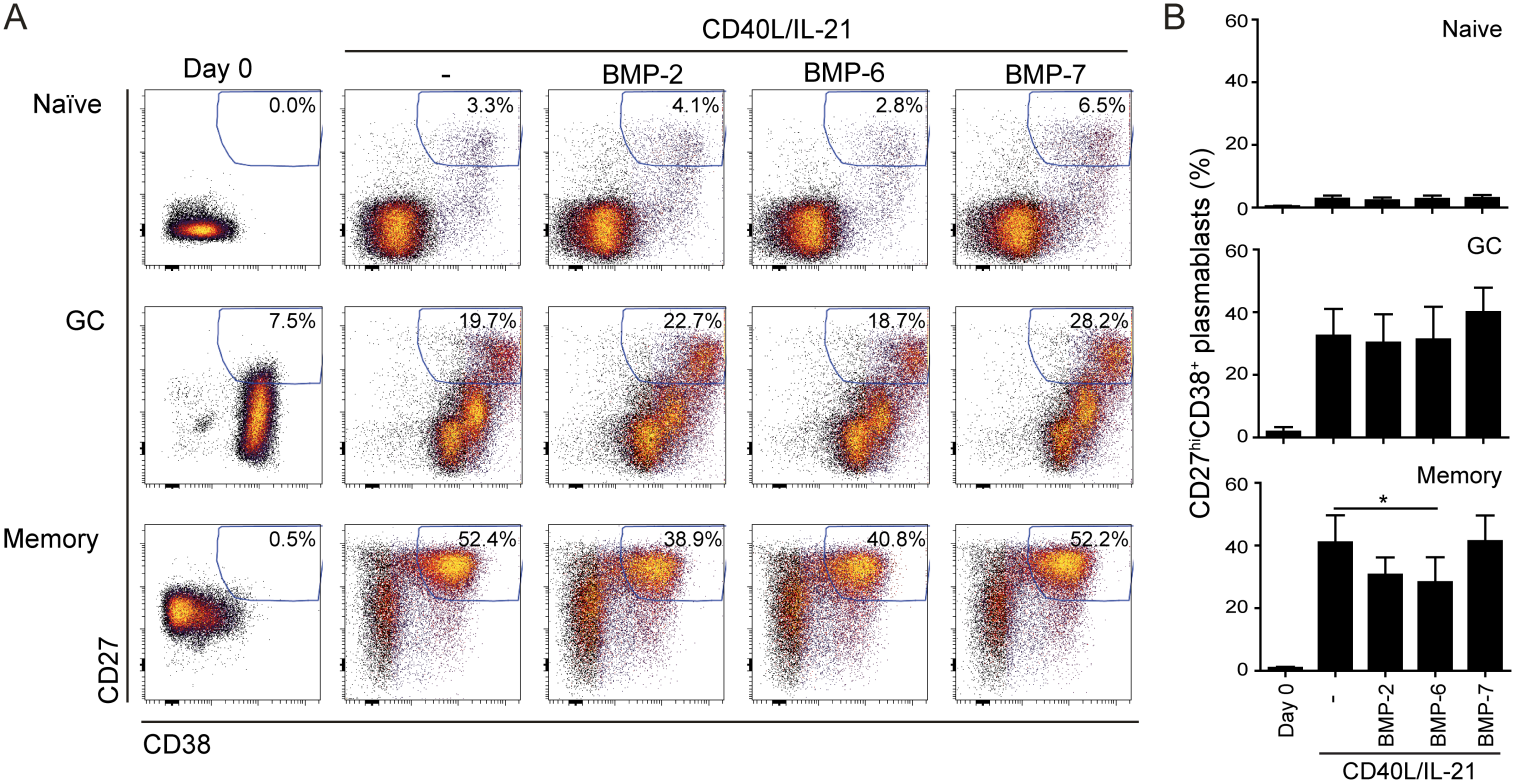
BMPs do not affect plasmablast differentiation in GC B cells. GC, naïve and memory B cells were isolated by FACS-sorting and cultured on a feeder layer of HK cells for four days in the presence of CD40L/IL-21 and different BMPs before analysis by flow cytometry. Plasmablasts are gated as CD38^+^CD27^hi^. Shown here is percentage of plasmablasts in (A) one representative experiment or as (B) mean ± SEM, *n* = 4 (GC), *n* = 5 (memory) *n* = 6 (naïve). Statistical testing was performed against CD40L/IL-21 condition. *p < 0.05; two-tailed Student’s t-test, unequal variance.

We next investigated whether BMPs could exert functional effects on *in vitro* cultured GC B cells. As these cells are highly prone to apoptosis, a co-culture system featuring the FDC-like HK cell line was introduced to improve viability [37, 38]. Addition of cytokines was required to promote plasmablast differentiation, and initial testing identified CD40L and IL-21 as the optimal combination (S2 Fig). Hence, FACS-sorted GC, naïve and memory B cells were then cultured on HK cells together with CD40L/IL-21 and BMP-2, -6 or -7 for 4 days. In line with our previous results [34], BMP-2 and -6 significantly suppressed CD40L/IL-21-induced plasmablast differentiation of memory B cells, but not of GC B cells (Fig 3). BMP-7 had no effect on plasmablast differentiation from GC B cells or memory B cells (Fig 3). The presence of HK cells could potentially counteract the effects of exogenously added BMPs, by producing BMPs and/or by expressing BMP receptors. However, with the exception of *BMP4*, endogenous *BMP* expression levels were low or undetectable (S3A Fig). Analysis of receptor expression revealed abundant expression of ALK2 but low expression of the other receptors (S3B Fig), indicating that HK cells could compete for exogenously added BMPs. To test if HK cells could influence BMP-induced effects in B cells, we used peripheral blood memory B cells, as they could be cultured without HK cells [34]. Culture of memory B cells in the absence of HK cells gave higher percentage of plasmablasts after 6 days, as compared to co-cultures with HK cells, but the inhibitory effects of BMP-6 and BMP-7 remained comparable (S3C Fig). These results suggest that CD40L/IL-21- induced plasmablast differentiation from GC B cells is not affected by BMPs, which contrasts the effects in memory B cells.

**Fig 4:**
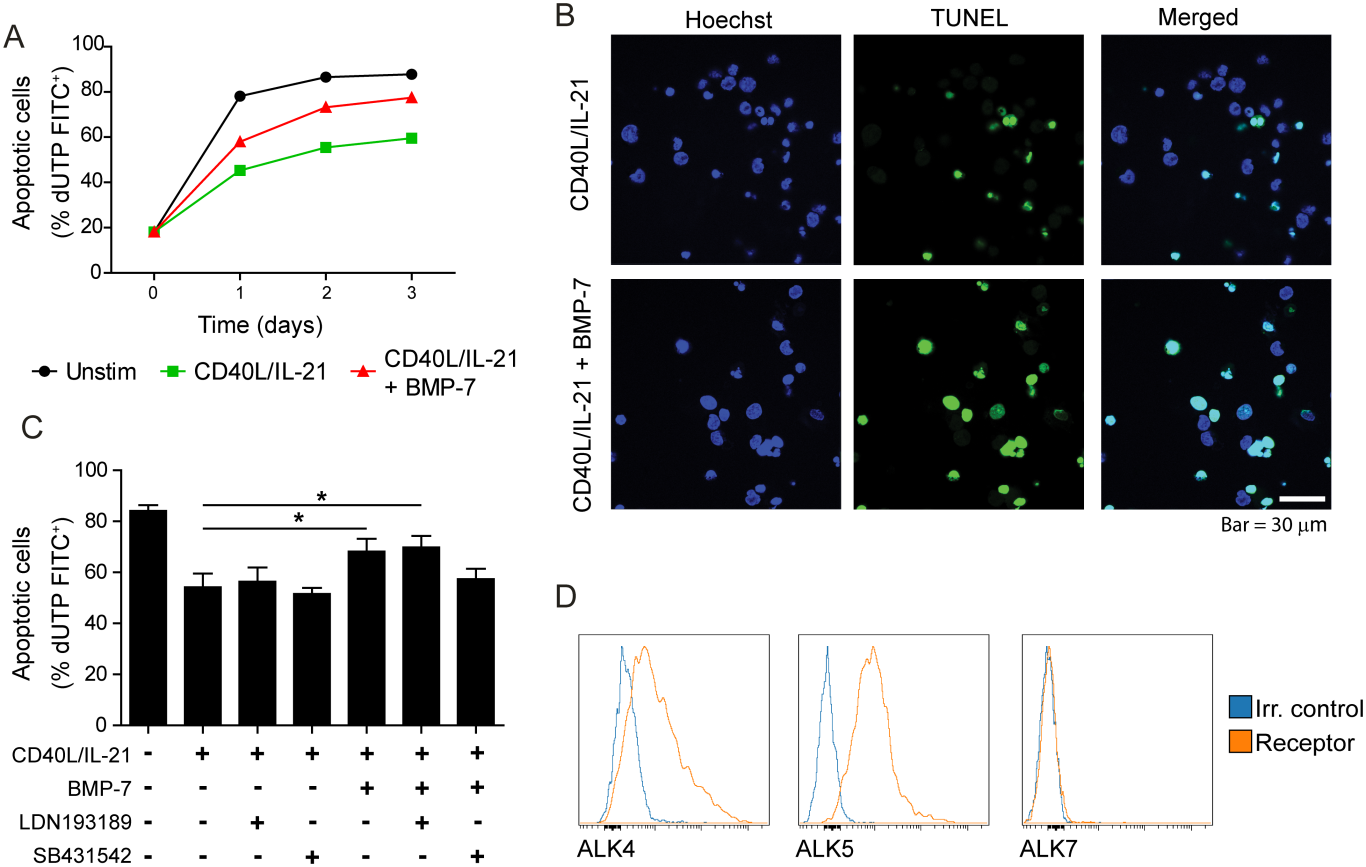
BMP-7 induces apoptosis in GC B cells and the effect is abrogated by selective inhibition of TGF-β type I receptor. (A-C) GC B cells were obtained by immunomagnetic bead separation and co-cultured with HK cells in the presence of CD40L/IL-21 with or without BMP-7 and apoptosis was measured by TUNEL assay. (A) Cells were cultured for up to 3 days and analyzed by flow cytometry. Shown is one representative of 2 donors. (B) After 2 days in culture, TUNEL staining (green) and Hoechst staining (blue) was detected by confocal microscopy. Representative images from one of three independent experiments are presented. Scale bar represents 30 μm. (C) The ALK 2/3 and ALK 4/5/7 selective inhibitors, LDN193189 and SB431542, respectively, were added to the cultures as specified and apoptosis was measured by flow cytometry at day 2. Mean ±SEM, *n* = 4, (D) Single cell suspensions from human tonsils were stained with lineage markers and anti-ALK4, anti-ALK5 or anti-ALK7 antibodies, and analyzed by flow cytometry. Shown are histogram overlays of receptor expression as compared to an irrelevant control. All experiments were repeated at least twice. Statistical testing was performed against CD40L/IL-21 condition. *p < 0.05; two-tailed, paired Student’s *t*-test.

We have previously demonstrated that naïve and memory B cells are highly prone to BMP-7-induced apoptosis [34]. To assess if BMP-7 also could induce cell death in GC B cells, they were cultured with HK cells together with CD40L/IL-21 in the presence or absence of BMPs for 4 days. Propidium iodide staining revealed that BMP-7 could counteract CD40L/IL-21-induced survival, whereas BMP-2, -4 and -6 had no effect (S4 Fig). TUNEL assay, detecting dUTP^+^ cells by flow cytometry demonstrated that BMP-7 induced apoptosis in GC B cells, and the effect was detectable after 1 day of culture (Fig 4A). Confocal microscopy, measuring dUTP^+^ apoptotic cells further confirmed these results (Fig 4B). Again, the presence of HK cells could potentially influence the effects of BMP-7-induced apoptosis. This was tested by culturing peripheral blood memory B cells in the presence of CD40L, with or without HK cells. Whereas the percentage of dead cells was greatly reduced in the presence of HK cells, the viability-suppressing effect of BMP-7 remained comparable (S3D Fig). This suggests that the BMP-7-induced effect was not confounded by the presence of HK cells.

### Truncated ALK5 but not truncated ALK4 can counteract BMP-7-induced apoptosis

To explore the mechanism for BMP-7-induced apoptosis in GC B cells, we next tested if this effect could be counteracted by the selective BMP receptor type I ALK2/ALK3 inhibitor LDN193189. Based on the knowledge of receptor promiscuity in the TGF-β superfamily, the TGF-β-receptor type I inhibitor SB431542, selective for ALK4/ALK5/ALK7, was also tested. GC B cells were cultured for two days with HK cells together with CD40L/IL-21 in the presence or absence of BMP-7, with or without inhibitors. BMP-7 significantly counteracted the viability-promoting effects of CD40L/IL-21 (*p* < 0.012, Fig 4C). BMP-7 in the presence of the ALK2/ALK3 inhibitor demonstrated an effect similar to BMP-7 (*p* < 0.017), while BMP-7 in the presence of the ALK4/ALK5/ALK7 inhibitor had no significant apoptosis-inducing effect (Fig 4C). Receptor analysis revealed that ALK4 and ALK5 were abundantly expressed in GC B cells whereas ALK7 was not detectable (Fig 4D), suggesting that induction of apoptosis by BMP-7 could be mediated through ALK4 or ALK5.

**Fig 5:**
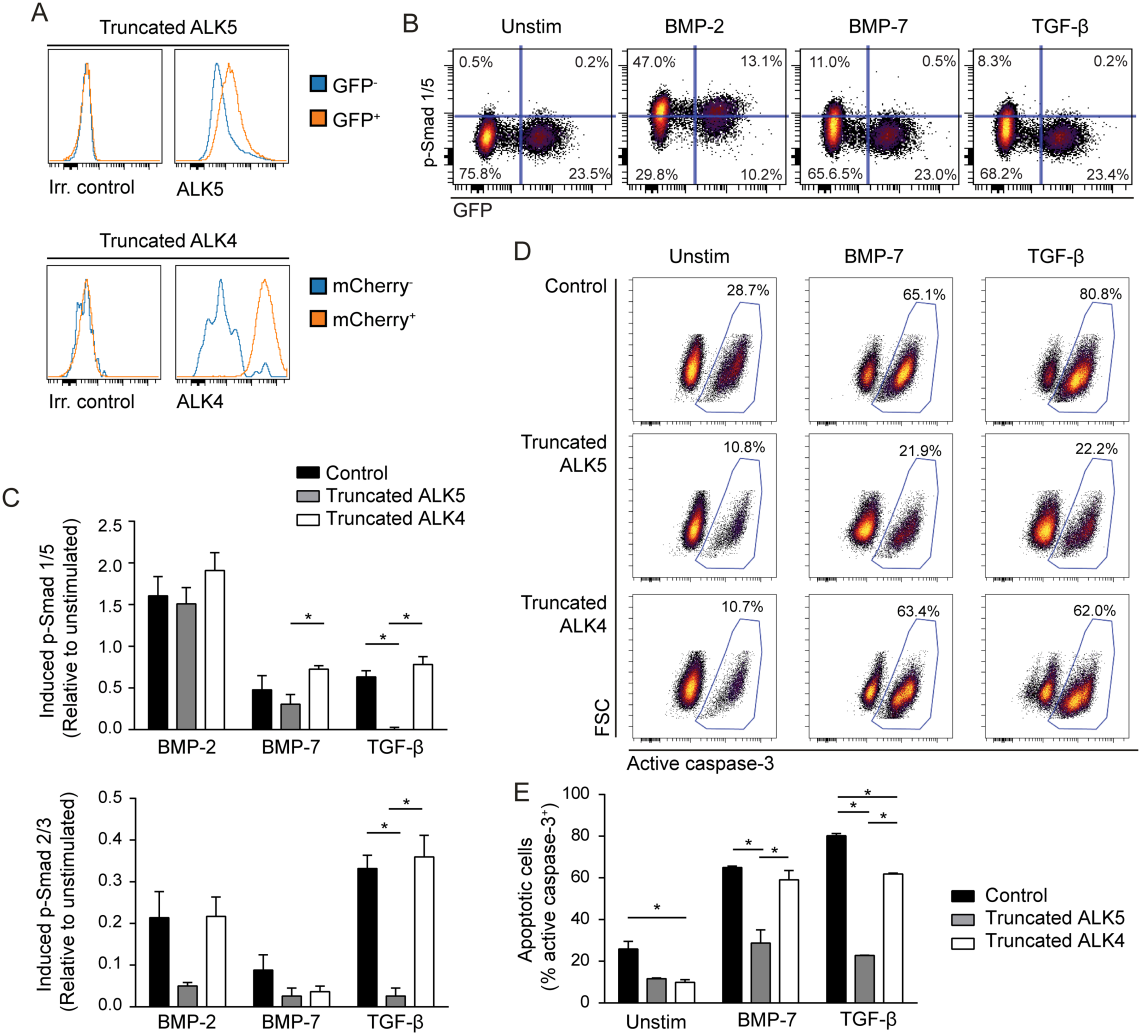
Introduction of truncated ALK5 in the B-cell lymphoma cell line Mino abrogates BMP-7-induced apoptosis. Mino cells were transduced with truncated ALK5 or truncated ALK4. (A) The cells were stained with biotinylated anti-ALK5, followed by streptavidin PE or by biotinylated anti-ALK4, followed by streptavidin APC, and analyzed by flow cytometry. Receptor expression is compared in GFP^+^ or mCherry^+^ transduced cells vs. GFP^-^/mCherry^-^ non-transduced cells. (B-C) The transduced Mino cells were cultured in serum free media (X-VIVO 15) over night and then left in medium alone (unstim) or stimulated with BMP-2, BMP-7 or TGF-β for 60 min, before detection of phosphorylated (p-) Smad 1/5 or p-Smad 2/3 by flow cytometry. (B) One representative experiment showing p-SMAD1/5 vs. GFP in truncated ALK5-2A-GFP expressing cells. (C) BMP- or TGF-β-induced phosphorylation is shown relative to unstimulated cells, using arcsinh transformation of median fluorescence intensity data. Mean ± SEM, *n* = 5. (D-E): Transduced Mino cells were cultured in X-VIVO 15 and left unstimulated or stimulated with TGF-β or BMP-7 for 72 hours and stained for active caspase-3 before analysis by flow cytometry. Shown here is active caspase-3 staining of control cells and transduced cells for (D) one representative experiment and (E) mean ± SEM, *n* = 3. *p < 0.05; two-tailed, paired Student’s *t*-test.

TGF-β-induced apoptosis is mediated via binding to ALK5 [19, 39], and BMP-7 showed similar signaling pattern to TGF-β in the B-cell lymphoma cell line Mino with phosphorylation of both Smad 1/5 and Smad 2/3, although at lower levels (S5 Fig). Mino expressed ALK5 and was highly sensitive to BMP-7-induced apoptosis (S5 Fig), in contrast to other B-cell lymphoma cell lines [35]. We further explored the mechanism for BMP-7-mediated apoptosis in Mino cells by overexpressing a GFP control vector, truncated ALK5 or truncated ALK4 (Fig 5A and S6 Fig), and then studied BMP-2, -7 or TGF-β-induced signaling. This demonstrated that expression of truncated ALK5 almost completely counteracted TGF-β-induced phosphorylation of both Smad 1/5 and Smad 2/3 as expected (Fig 5B and 5C). BMP-7-induced p-SMAD1/5 was also significantly reduced in cells with truncated ALK5 as compared to truncated ALK4, while the difference to control cells was not significant (Fig 5B and 5C). In comparison, BMP-2- induced p-SMAD1/5 was not affected in cells expressing the truncated receptors. Also note that truncated ALK4 had the expected effect upon Activin A-induced signaling (S7 Fig). To test if truncated ALK4 or ALK5 receptors could counteract BMP-7-induced apoptosis, Mino cells expressing truncated ALK5 or truncated ALK4 (GFP^+^ or mCherry^+^ FACS-sorted cells, respectively) or non-manipulated Mino cells were cultured with or without TGF-β or BMP-7 for 3 days before detection of apoptotic cells. This approach demonstrated that the presence of truncated ALK5 almost completely counteracted BMP-7- and TGF-β-induced apoptosis (Fig 5D and 5E, S6 Fig). In contrast, truncated ALK4 had no effect on BMP-7-induced apoptosis, but had a partial reduction on TGF-β-induced apoptosis. This could be due to antagonism of endogenous TGF-β superfamily ligand(s), as percentage of apoptotic cells also was reduced in the absence of ligands. Together, these findings suggest that expression of ALK5 can influence BMP-7 effects.

### GC B cells express BMP-7

**Fig 6:**
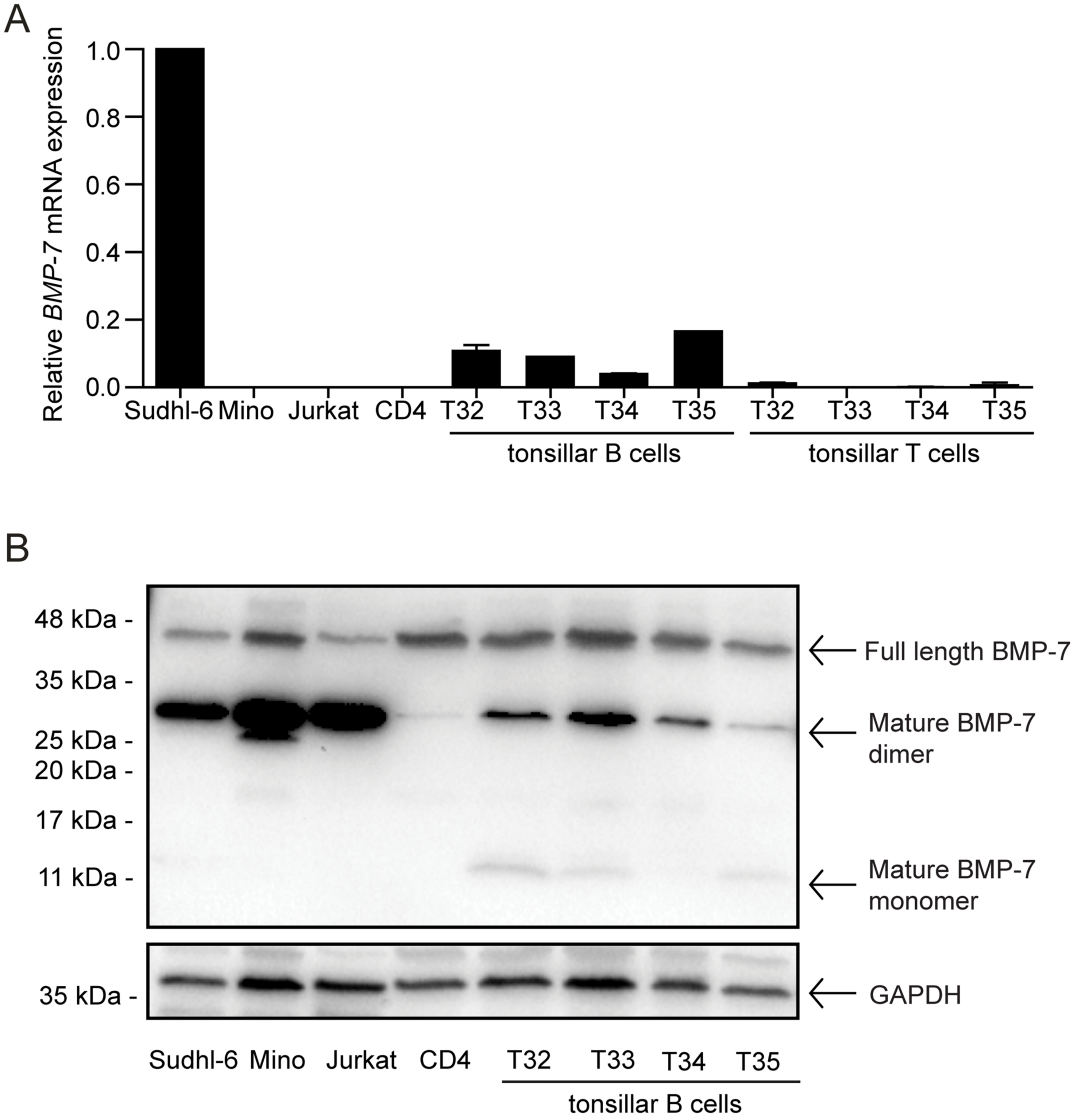
BMP-7 is present in tonsillar B cells. Primary tonsillar B- and T cells were obtained by immunomagnetic bead isolation, by using positive selection for T cells (CD3 Dynabeads) and the remaining negatively selected cells are mainly B cells. (A) Gene expression of BMP7 was determined by qPCR duplicates and is shown relative to PGK-1 and GAPDH endogenous control and normalized to Sudhl-6. Median ± SD, *n* = 2. (B) Protein levels of BMP-7 were determined by western blotting. Included were cell lines Sudhl-6, Mino and Jurkat, CD4^+^ T cells from peripheral blood, tonsillar B cells from four donors (denoted T32-35).

We have previously demonstrated expression of *BMP7* mRNA in GC B cells, separated into centroblasts and centrocytes [35]. To further confirm these previous findings, we isolated B and T cells from 4 tonsil donors. Tonsillar B cells from all donors had detectable *BMP7* mRNA, although at lower levels than the B-cell lymphoma cell line Sudhl-6 (Fig 6A). In contrast, T cells in general showed little or no detectable *BMP7*. This was shown with 3 different probes targeting different parts of the BMP-7 mRNA (S8 Fig). Western blot analysis confirmed that BMP-7 protein was expressed in tonsillar B cells, both as mature monomers and dimmers, and as propeptide (Fig 6B). The presence of BMP-7 in tonsillar B cells shows that it is physiological relevant to study BMP-7-induced effects in B cells undergoing maturation and selection in the GC.

## Discussion

The expression pattern of BMPs has not been fully explored in secondary lymphoid structures. However, our finding that BMP-7 is present in tonsillar B cells demonstrates that it is physiological relevant to study BMP-7-induced effects in B cells undergoing maturation and selection in the GC. We and others have demonstrated expression of *BMP7* mRNA in GC B cells from human tonsils and in malignant B cells from follicular lymphoma (FL) [35] and mantle cell lymphoma (MCL) patients [40]. FL and MCL are malignancies originating from GC and mantle zone non-GC B cells, respectively. Hence, the results presented here as well as these prior studies suggest that BMP-7 can be produced by normal as well as malignant GC B cells. In the current study, we show that BMP-7 counteracted the viability-promoting effect of CD40L in GC B cells. The TGF-β receptor type I-selective inhibitor SB431542, but not by the BMP receptor type I-selective inhibitor LDN 193189 diminished the BMP-7-induced effects. Expression of truncated ALK5, lacking the kinase domain, in a BMP-7-sensitive B-cell line counteracted BMP-7-induced signaling and apoptosis. These results suggest that expression of TGF-β receptor ALK5 indirectly can influence BMP-7 binding to its specific receptors, and identifies BMP-7 as a negative regulator of GC B-cell survival, possibly in an autocrine manner.

BMP-7 has been shown to induce apoptosis in naïve and memory B cells from peripheral blood [34], as well as in myeloma cell lines and myeloma cells from patients [32]. BMP-7-induced apoptosis has also been described in other tissues as part of the normal development and homeostasis [41, 42]. On the contrary, BMP-7 can improve viability in some cell types [43, 44]. The apoptosis-inducing effect mainly seems to be mediated through the mitochondrial pathway as various BMPs, including BMP-7, have been shown to act via activation of caspase-3, -8 and -9 [41, 42, 45] and/or via downregulation of Bcl-2 [42]. Our data are in agreement with this as BMP-7 greatly increased the level of active caspase-3 in the B-cell lymphoma line Mino. Hence, although BMP-7-induced effects are highly context- and cell type dependent, this cytokine has a viability suppressive role at several stages of B-cell development, including GC B cells.

Our data demonstrated that the apoptosis-inducing effect of BMP-7 could be counteracted by overexpression of truncated ALK5, but not by truncated ALK4 in Mino cells, suggesting that BMP-7 mediates its effects via ALK5. However, we were not able to show binding between BMP-7 and ALK5 (not shown), in line with previous data demonstrating that BMP-7 could bind to ALK2 and ALK3, but not to ALK5 [46, 47]. Based on this, we hypothesize that overexpression of truncated ALK5 in Mino cells might influence BMP-7-induced apoptosis indirectly by outcompeting other BMP type I receptors for complex formation with type II receptors, as has recently been demonstrated for ALK2 on BMP2/4-ALK3 mediated effects [48]. Still, in primary GC B cells, an ALK4/5/7 inhibitor counteracted BMP-7-induced apoptosis, further supporting a role of ALK5 as mediator for BMP-7 effects. Crosstalk between canonical BMP and TGF-β receptors has been seen previously, as BMP-2 could induce complex formation between BMPRII and ALK5 or ALK7 [19]. Furthermore, BMPs can signal through the TGF-β-associated Smad2/3 pathway as demonstrated in the current study and by others who demonstrated BMP-2, -4, -6 and -7-induced phosphorylation of Smad 2 and Smad 3 in transformed cell lines [19]. Importantly, by using selective ALK5 inhibitors or *ALK5* mutants, this receptor has been indicated to mediate TGF-β-induced apoptosis [49, 50]. In human GC B cell centroblasts, TGF-β accelerated spontaneous apoptosis whereas the presence of the ALK4/ALK5/ALK7-selective inhibitor SB431542 alone counteracted this [51]. Therefore, the authors speculated that TGF-β was involved in autocrine signaling [51]. Furthermore, we have previously shown that TGF-β sensitive B-lymphoma cell lines express high levels of ALK5 [52]. Together, these observations suggest that ALK5 signaling has a homeostatic role in GC B cells.

In the present study, we show that the combination of CD40L and IL-21 potently induced differentiation of GC B cells to CD38^+^CD27^hi^ plasmablast as demonstrated by us and others [7, 9, 34]. The addition of BMP-2 and BMP-6 suppressed plasmablast differentiation from memory B cells, but did not affect the differentiation process of GC B cells. This might in part be due to the low expression of ActRIIb in GC B cells vs. memory B cells. The activin type II receptors are high affinity receptors for activins, but are also low affinity receptors for BMPs, including BMP-2 and BMP-7 [53, 54]. The low expression or absence of a low-affinity type II receptor could be sufficient to abrogate effects commonly mediated via ActRIIb complexes. Furthermore, the use of HK cells to support the survival of GC B cells could potentially affect BMP-induced effects in our study. HK cells are similar to early-stage FDCs and secrete various B-cell growth factors, including IL-6 and Notch [37, 38, 55]. Crosstalk between the Notch- and BMP-pathway has been shown [56]. In addition, our data showed that the HK cells had detectable level of ALK2 and expressed *BMP4* mRNA. Hence, HK cells could potentially influence the co-culture of GC B cells by production of BMPs and by competing for exogenously added BMPs. However, experiments with memory B cells cultured with or without HK cells did not indicate that HK cells interfered with BMP-induced effects.

In conclusion, our work identified BMP-7 as a negative regulator of GC B-cell survival, hence adding another component to the regulation of B cells undergoing GC reaction. Our data suggests that BMP-7-induced apoptosis could be influenced by the TGF-β receptor ALK5 in an indirect manner, further demonstrating the receptor complexity within the TGF-β superfamily.

## Supporting information

S1 Table qPCR probes and target genes. (PDF)

S1 Fig Immunomagnetic bead isolation of GC and naïve/memory B-cell subsets. (PDF)

S2 Fig The viability of GC B cells is improved by co-culturing with HK cells and with CD40L/IL-21. (PDF)

S3 Fig BMP and BMPR expression in the HK cell line could influence BMP effects, but BMP-induced effects in memory B cells remains comparable in the presence or absence of HK cells. (PDF)

S4 Fig BMP-7 induces cell death in GC B cells. (PDF)

S5 Fig The B-cell lymphoma cell line Mino expresses Alk5 and is highly sensitive to BMP-7-induced apoptosis. (PDF)

S6 Fig Effects of BMP-7 in Mino cells expressing truncated ALK5. (PDF)

S7 Fig Activin A-induced signaling in cells overexpressing FL ALK4 or truncated ALK4. (PDF)

S8 Fig Relative mRNA expression of BMP-7 in tonsillar B cells. (PDF)
