# Supplementary material for: BMP-7 induces apoptosis in human germinal center B cells and is influenced by TGF-β receptor type I ALK5
Source: PLoS One. 2017 May 10;12(5):e0177188. doi: 10.1371/journal.pone.0177188 (PMC5425193; doi:10.1371/journal.pone.0177188)
Supplement: S1 Table — (PDF) [file pone.0177188.s001.pdf]

Supplemental table 1: qPCR probes and target sequences

Assay ID	Target	Sequence
Hs00195432_m1	*SMAD1*	gatacgcccc cacctgctta cctgcctcct gaagacccca tgacccagga tggctctcag ccgatgg
Hs00195437_m1	*SMAD5*	atctcc aaacagccct tatcccctt ctcctgctag cagcacatat cccaactccc cagcaagttc tggaccagga agtccatttc agctcccagc
Hs00929647_m1	*SMAD4*	caaggtt gcacataggc aaaggtgtgc agttggaatg taaaggtgaa ggtgatgttt gggtcaggtg ccttagtgac cacgcggtct
Hs00178579_m1	*SMAD6*	ggatctgt ccgattccac attgtcttac actgaaacgg aggctaccaa ctccctcatc actgctccgg gtgaattctc agacgccagc atg
Hs00998193_m1	*SMAD7*	ccaacttctt ctggagcctg gggatcggtc acactggtgc gtggtggcat actgggagga gaagacgaga gtggggaggc tctactgtgt ccaggagccc tctctg
Hs03676575_s1	*ID1*	cg ccggatctga gggagaacaa gaccgatcgg cggccactgc gcccttaact gcatccagcc tggggctgag gctgaggcac tggcgaggag agggcgct
Hs04187239_m1	*ID2*	ggtgtt catgatttct tttattcttt gcacaacaac aacaacaaca aattcacgga atcttttaag tgctgaactt attttt
Hs00954037_g1	*ID3*	cagaacgcag gtgctggcgc ccgttctgcc tgggaccccg ggaacctctc ctgccggaag ccggacggca gggatgggcc ccaacttcgc cctgcccact tgacttcacc aaatcccttc ctggagact
Hs00233476_m1	*BMP7*	ag ggaatcggat ctcttcctgc tcgacagccg taccctctgg gcctcggagg agggctggct ggtgtttgac atc
