# Supplementary material for: BMP-7 induces apoptosis in human germinal center B cells and is influenced by TGF-β receptor type I ALK5
Source: PLoS One. 2017 May 10;12(5):e0177188. doi: 10.1371/journal.pone.0177188 (PMC5425193; doi:10.1371/journal.pone.0177188)
Supplement: S1 Fig — (PDF) [file pone.0177188.s002.pdf]

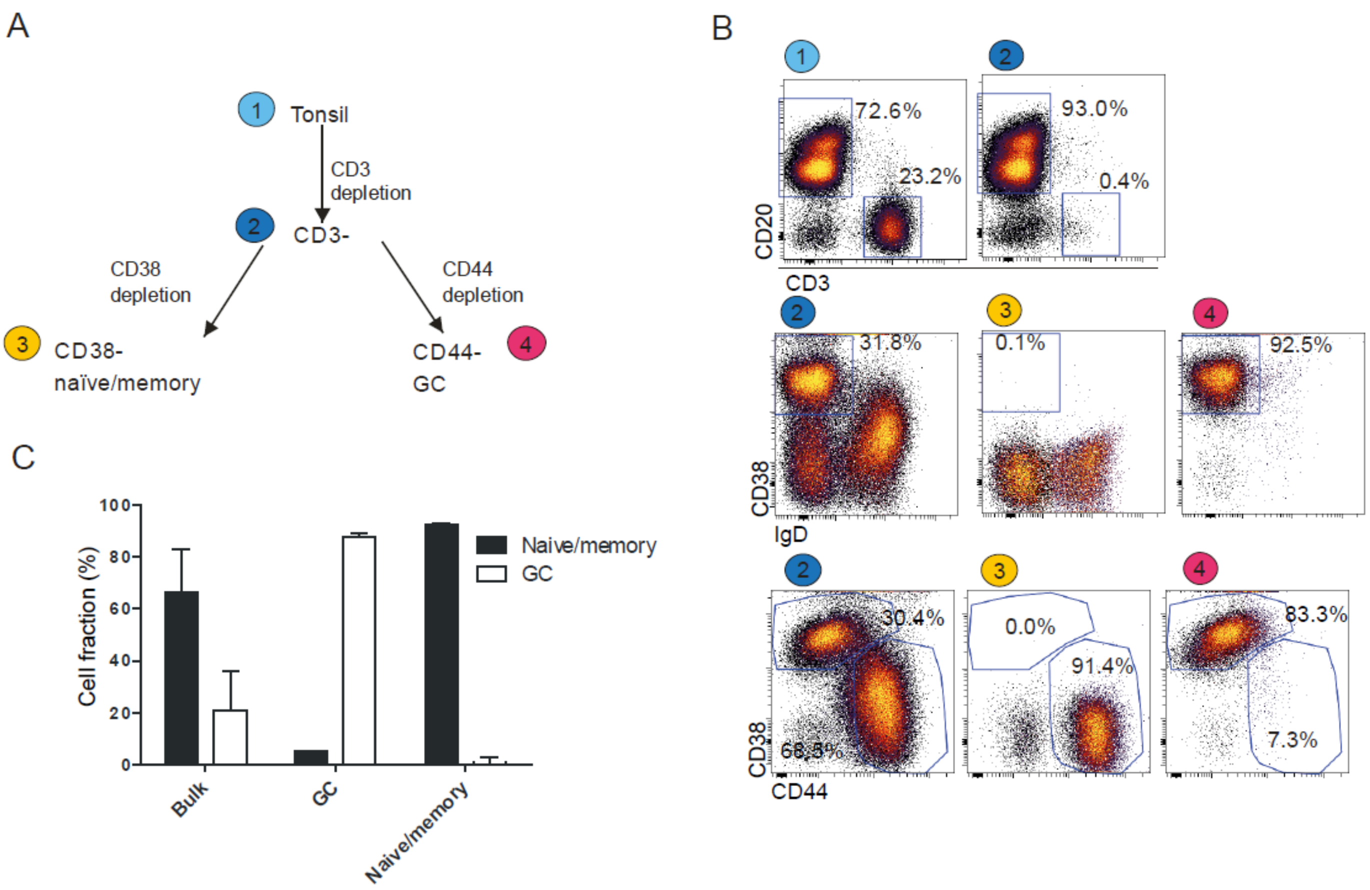

Supplemental Figure 1

Immunomagnetic bead isolation of GC and naïve/memory B-cell subsets.

Single cells from tonsils were thawed and depleted for $CD3^{+}$ T cells followed by either CD44 or CD38 depletion, using immunomagnetic beads. (A) The experimental pipeline. (B) Cells from the different isolation steps, labeled 1-4 according to populations in A, were stained with surface markers and analyzed by flow cytometry. The first row shows CD3-depletion efficacy. The middle row shows depletion efficacy with respect to expression of CD38 and IgD. The lower row shows depletion efficacy with respect to expression of CD38 and CD44. GC B cells were identified as $CD20^{+}CD38^{+}IgD^{-}CD44^{-}$, and naive/memory B cells as $CD20^{+}CD38^{low}CD44^{+}$. (C) Shown here is the enrichment of GC B cells or naive/memory B cells in bulk (before bead selection) and after final selection steps for GC or naive memory B cells, mean ± SEM, $n = 3$.
