# Supplementary material for: BMP-7 induces apoptosis in human germinal center B cells and is influenced by TGF-β receptor type I ALK5
Source: PLoS One. 2017 May 10;12(5):e0177188. doi: 10.1371/journal.pone.0177188 (PMC5425193; doi:10.1371/journal.pone.0177188)
Supplement: S2 Fig — (PDF) [file pone.0177188.s003.pdf]

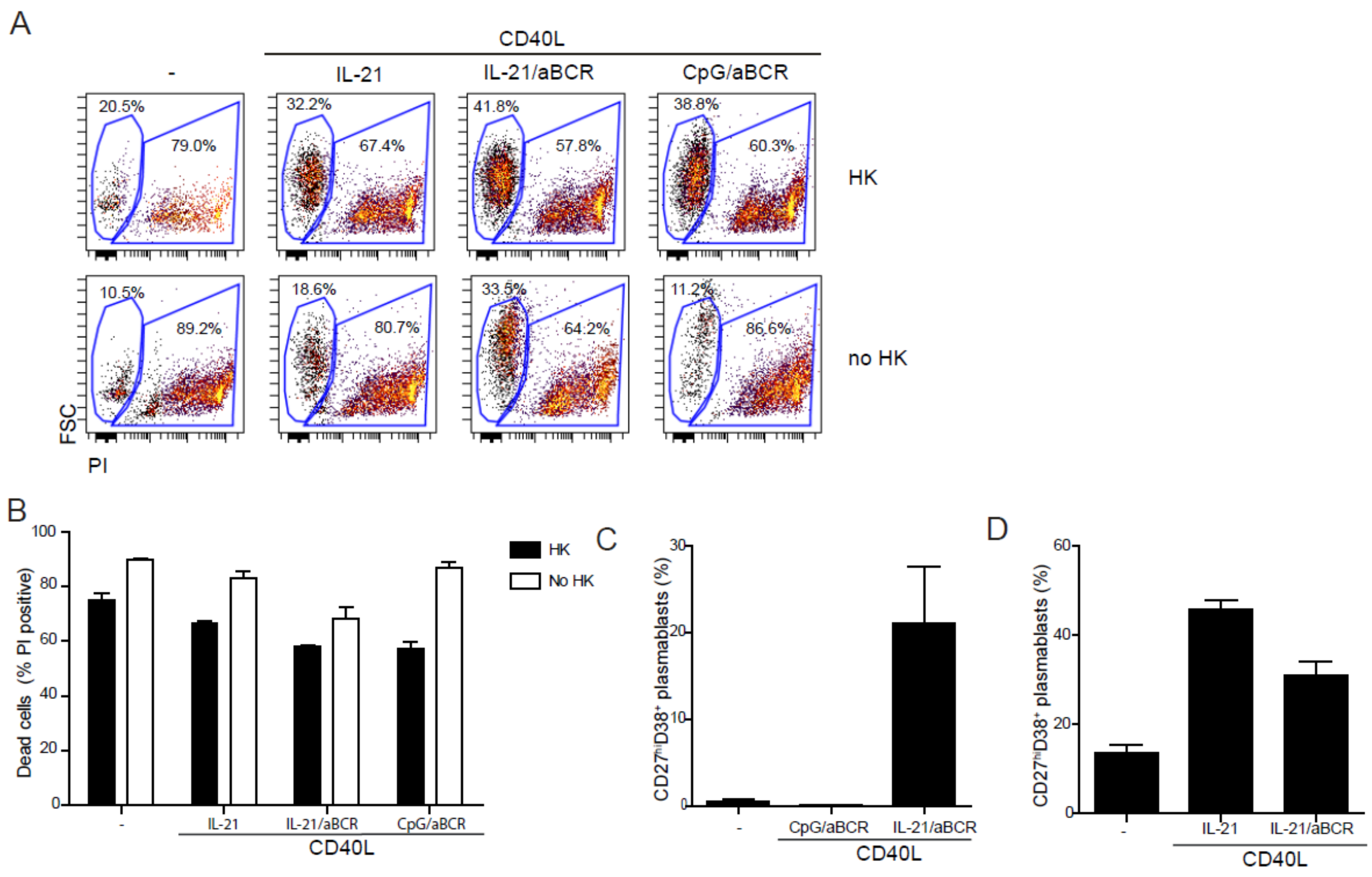

Supplemental Figure 2

The viability of GC B cells is improved by co-culturing with HK cells and with CD40L/IL-21.

GC B cells were obtained from tonsillar single cell suspension by using immunomagnetic beads as described in Supplemental Fig 1. The cells were stimulated with different cytokine cocktails as indicated and grown with or without HK stroma cells for four days followed by PI staining or immuno-phenotyping and analysis by flow cytometry. (A) Shown is dead cells determined by PI+ cells in one out of two experiments, and (B) mean ± SEM, n=2. (C,D) Bead-isolated GC B cells were cultured for six days in the presence of HK cells and with additional stimulation as specified before staining with antibodies to identify plasmablasts by flow cytometry analysis. Shown are $CD27^{hi}CD38^{+}$ plasmablasts, mean ±SEM, n = 3.
