# Supplementary material for: BMP-7 induces apoptosis in human germinal center B cells and is influenced by TGF-β receptor type I ALK5
Source: PLoS One. 2017 May 10;12(5):e0177188. doi: 10.1371/journal.pone.0177188 (PMC5425193; doi:10.1371/journal.pone.0177188)
Supplement: S3 Fig — (PDF) [file pone.0177188.s004.pdf]

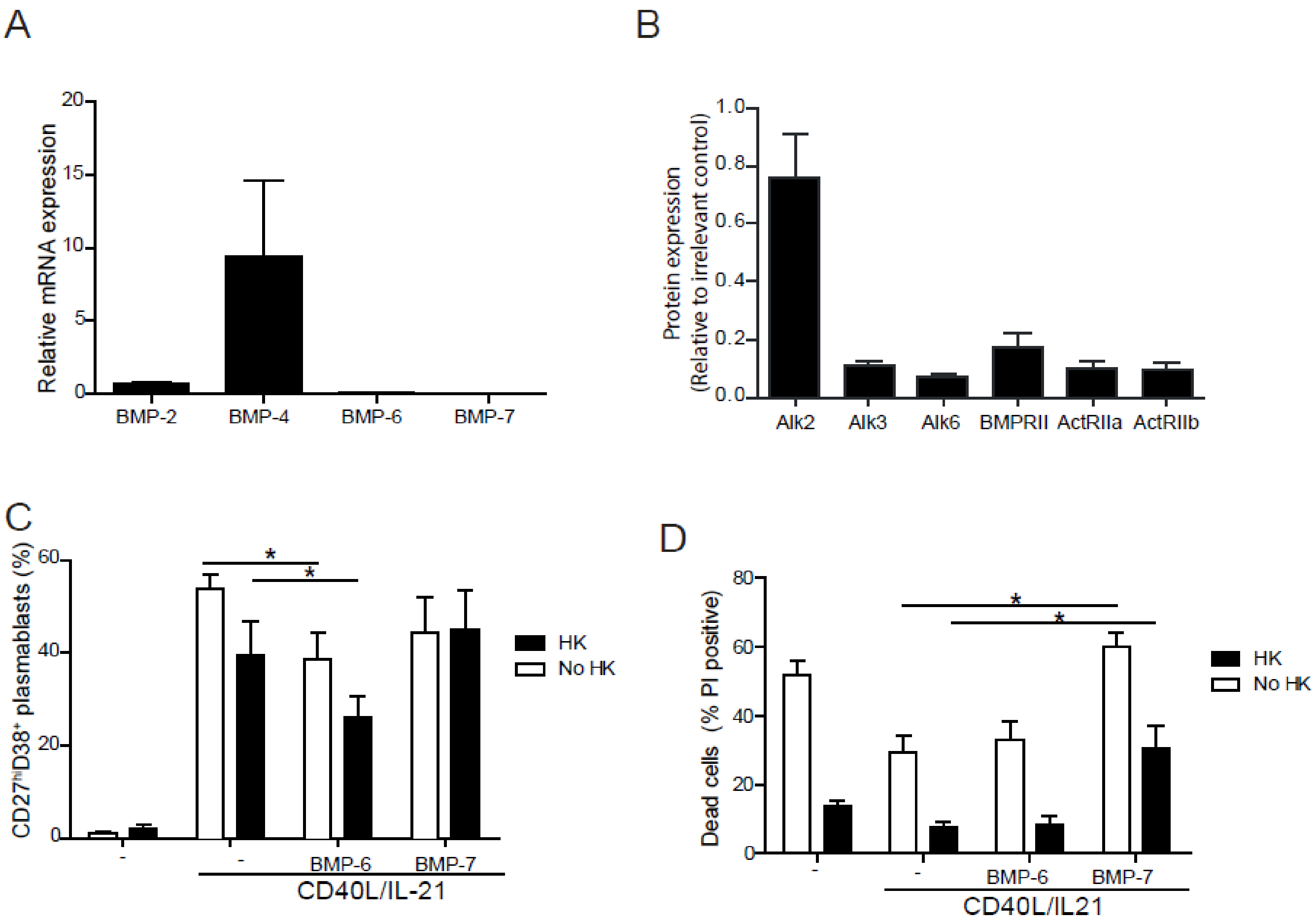

Supplemental Figure 3

BMP and BMPR expression in the HK cell line could influence BMP effects, but BMP-induced effects in memory B cells remains comparable in the presence or absence of HK cells.

(A) RNA was isolated from the HK cell line and analyzed by qPCR. The samples were run in singleplex with PGK-1 as endogenous control Relative mRNA expression was calculated by using the ΔΔCT-method, and normalized to the level in human fetal brain, mean ± SD, *n* = 2. (B) The HK cell line was stained with biotinylated antibodies, followed by streptavidin PE and flow cytometry analysis. Shown here are arcsinh transformed data, relative to irrelevant control, mean ± SEM, *n* = 3. (C,D) IgD$^-$ memory B cells from peripheral blood were obtained using immunomagnetic beads and cultured for 6 days before assessing (C) plasmablast differentiation or (D) viability (PI-staining) by flow cytometry. Shown here are mean ± SEM, *n* = 4. * denotes statistical significance calculated by a paired, two-tailed Students's *t*-test.
