# Supplementary material for: BMP-7 induces apoptosis in human germinal center B cells and is influenced by TGF-β receptor type I ALK5
Source: PLoS One. 2017 May 10;12(5):e0177188. doi: 10.1371/journal.pone.0177188 (PMC5425193; doi:10.1371/journal.pone.0177188)
Supplement: S4 Fig — (PDF) [file pone.0177188.s005.pdf]

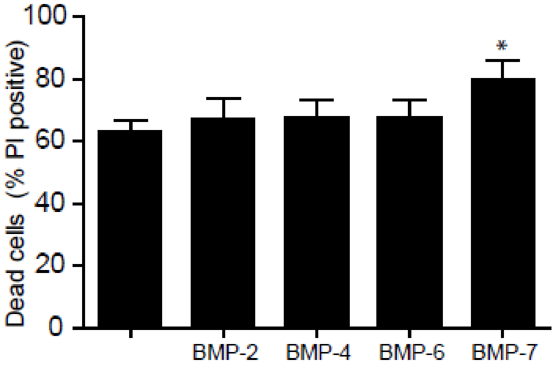

Supplemental Figure 4

BMP-7 induces cell death in GC B cells.

Bead-isolated GC B cells from human tonsils were seeded in 48-well plates pre-coated with HK cells and cultured for four days. The cells were then stained with (PI) and analyzed by flow cytometry. Shown here are percent PI+ cells, mean ± SEM, $n = 3$. * denotes statistical significance ($p < 0.05$), paired, two-tailed Students's *t*-test.
