# Supplementary material for: BMP-7 induces apoptosis in human germinal center B cells and is influenced by TGF-β receptor type I ALK5
Source: PLoS One. 2017 May 10;12(5):e0177188. doi: 10.1371/journal.pone.0177188 (PMC5425193; doi:10.1371/journal.pone.0177188)
Supplement: S5 Fig — (PDF) [file pone.0177188.s006.pdf]

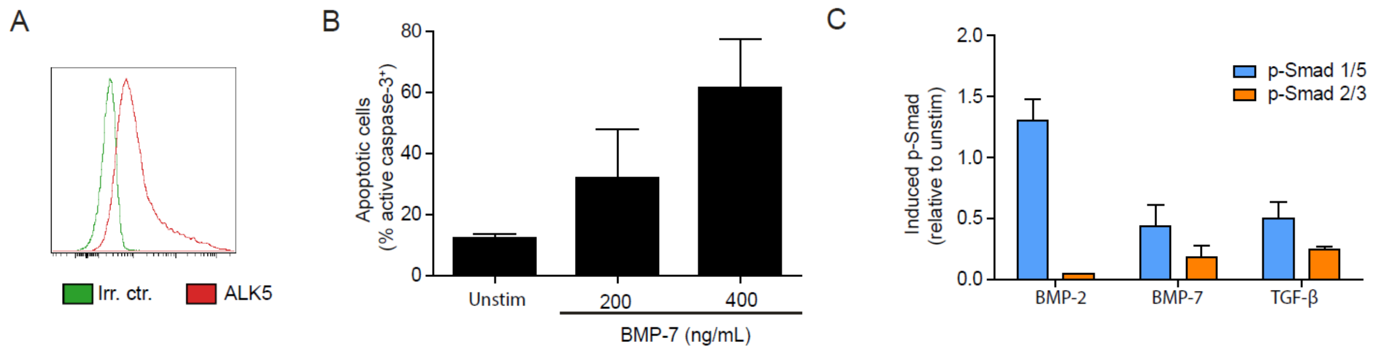

Supplemental Figure 5

The B-lymphoma cell line Mino expresses ALK5 and is highly sensitive to BMP-7-induced apoptosis

(A) Mino cells were stained with biotinylated anti-ALK5, followed by streptavidin PE and analyzed by flow cytometry. Shown here is ALK5 expression in Mino cells. (B) Mino cells were stimulated with BMP-7 for 72 hours and stained for active caspase-3 before analysis by flow cytometry. Shown is percent active caspase-3+ apoptotic cells, mean ± SEM, n= 3. (C) Mino cells were kept unstimulated or stimulated with BMP-2, BMP-7 or TGF-β for 60 min before fixation and permeabilization. The cells were then stained with phospho-specific antibodies for Smad 1/5 or Smad 2/3 and analyzed by flow cytometry. Shown here are phosphorylation levels in stimulated cells, relative to unstimulated cells, mean ± SD, n = 2.
