# Supplementary material for: BMP-7 induces apoptosis in human germinal center B cells and is influenced by TGF-β receptor type I ALK5
Source: PLoS One. 2017 May 10;12(5):e0177188. doi: 10.1371/journal.pone.0177188 (PMC5425193; doi:10.1371/journal.pone.0177188)
Supplement: S6 Fig — (PDF) [file pone.0177188.s007.pdf]

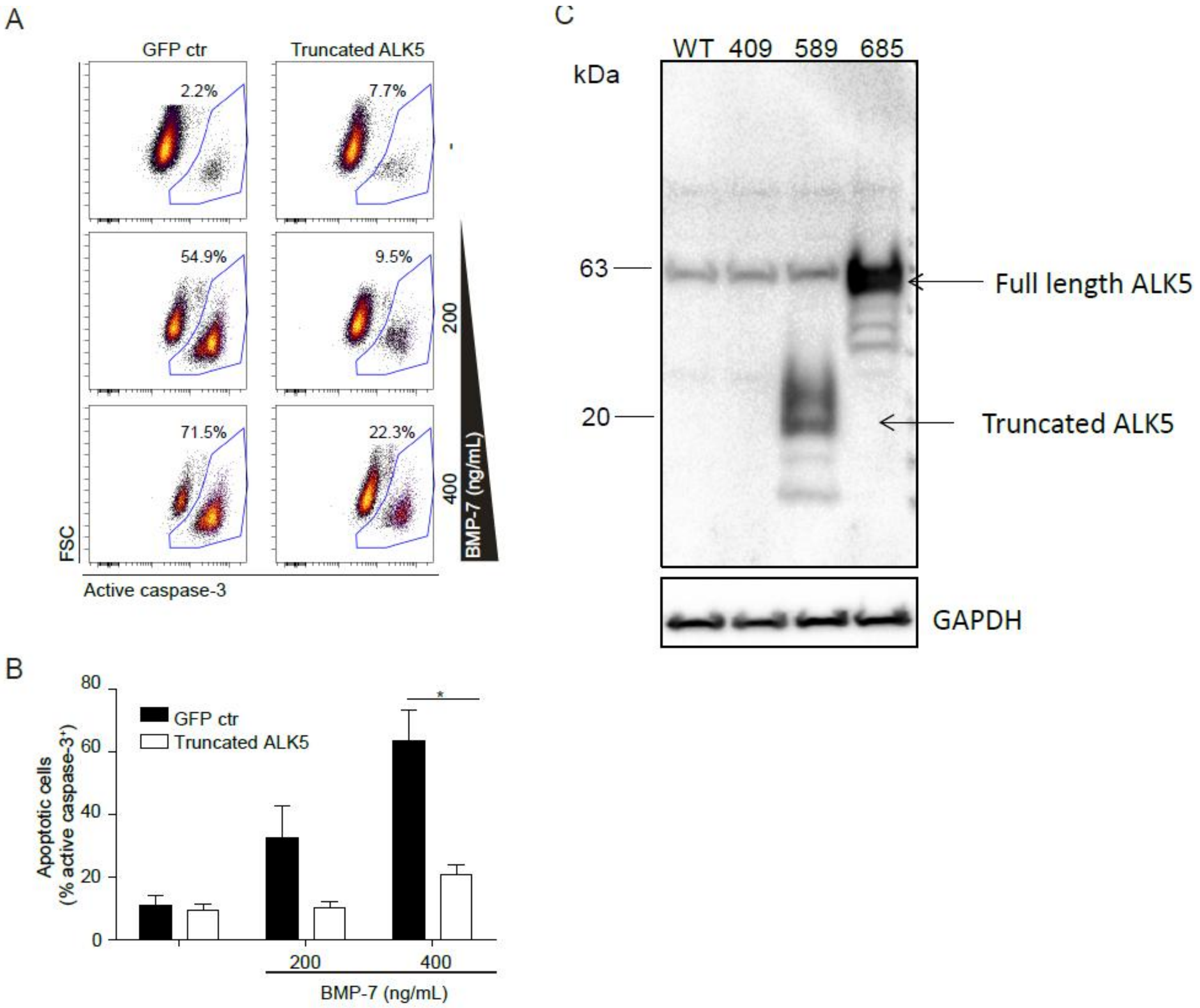

Supplemental Figure 6

Effects of BMP-7 in Mino cells expressing truncated ALK5.

Mino cells were transduced with GFP control or with truncated ALK5. Transduced Mino cells were cultured in X-VIVO 15 and left unstimulated or stimulated with BMP-7 (200 or 400 ng/ml) for 72 hours and stained for active caspase-3 before analysis by flow cytometry. (A) One representative experiment and (B) mean ± SEM, $n = 3$. (C) Detection of full length ALK5 or truncated ALK5 in Mino cells: WT:original cells, 409: GFP control, 589: truncated ALK5, 685: full lenght ALK5. * $p < 0.05$; two-tailed, paired Student’s *t*-test.
