# Supplementary material for: BMP-7 induces apoptosis in human germinal center B cells and is influenced by TGF-β receptor type I ALK5
Source: PLoS One. 2017 May 10;12(5):e0177188. doi: 10.1371/journal.pone.0177188 (PMC5425193; doi:10.1371/journal.pone.0177188)
Supplement: S7 Fig — (PDF) [file pone.0177188.s008.pdf]

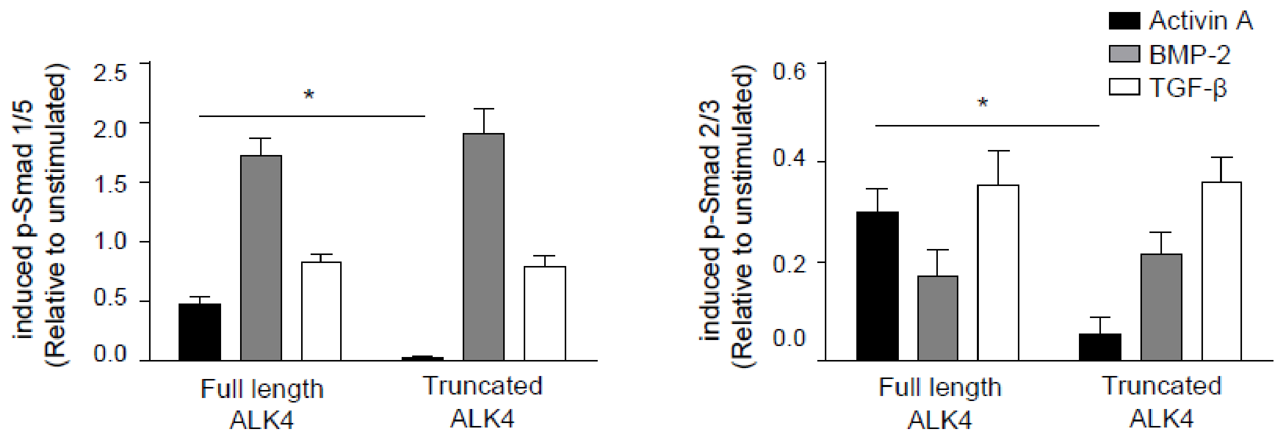


## Supplemental Figure 7

**Activin A-induced signaling in cells overexpressing FL ALK4 or truncated ALK4.**

Mino cells were transduced with full length or truncated ALK4. The transduced cells were cultured in serum free media (X-VIVO 15) over night and then left in medium alone (unstim) or stimulated with Activin A, BMP-2, or TGF-β for 60 min, before detection of phosphorylated (p-) Smad 1/5 or p-Smad 2/3 by flow cytometry. Induced phosphorylation is shown relative to unstimulated cells, using arcsinh transformation of median fluorescence intensity data. Mean ± SEM, $n = 3$. * $p < 0.05$; two-tailed, paired Student’s *t*-test.
