# Supplementary material for: BMP-7 induces apoptosis in human germinal center B cells and is influenced by TGF-β receptor type I ALK5
Source: PLoS One. 2017 May 10;12(5):e0177188. doi: 10.1371/journal.pone.0177188 (PMC5425193; doi:10.1371/journal.pone.0177188)
Supplement: S8 Fig — (PDF) [file pone.0177188.s009.pdf]

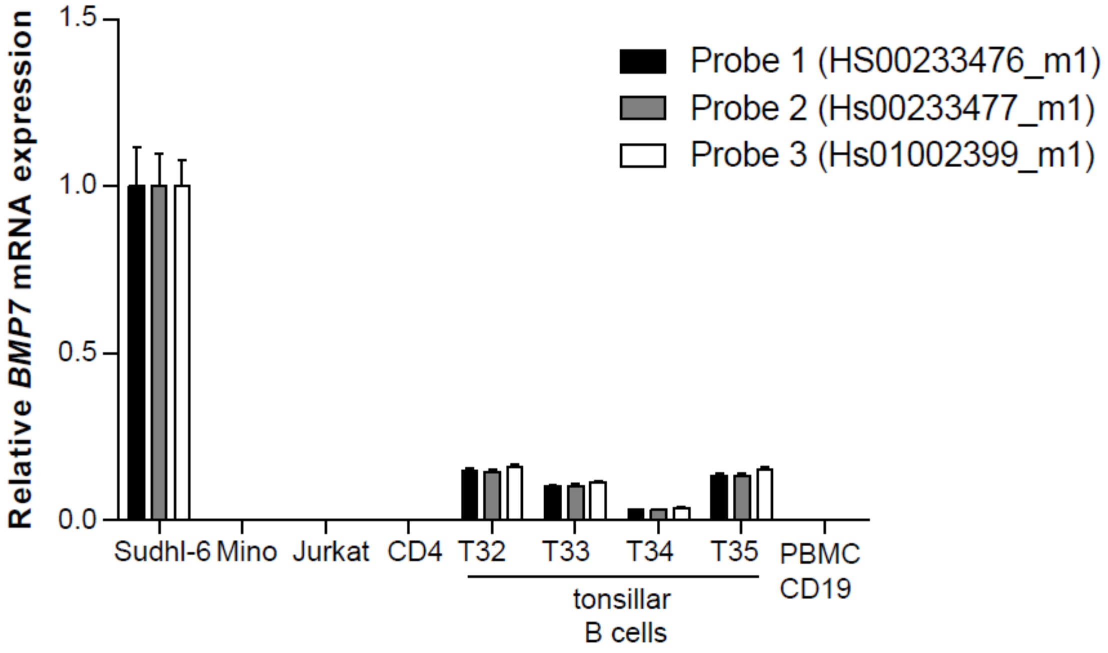

Supplemental Figure 8

Relative mRNA expression of BMP-7 in tonsillar B cells.

Primary tonsillar B cells were obtained by immunomagnetic bead isolation by negative selection for T cells (CD3 Dynabeads). Gene expression of BMP7 was determined by qPCR duplicates for 3 different probes and is shown relative to PGK-1 and GAPDH endogenous control and normalized to Sudhl-6. Mean ± SD.
